# Gastrospheres as a Model of Gastric Cancer Stem Cells Skew Th17/Treg Balance toward Antitumor Th17 Cells

**DOI:** 10.1155/2020/6261814

**Published:** 2020-12-23

**Authors:** Alaleh Rezalotfi, Ghasem Solgi, Marzieh Ebrahimi

**Affiliations:** ^1^Department of Immunology, School of Medicine, Hamadan University of Medical Sciences, Hamadan, Iran; ^2^Department of Stem Cells and Developmental Biology, Cell Science Research Center, Royan Institute for Stem Cell Biology and Technology, ACECR, Tehran, Iran; ^3^Psoriasis Research Center, Hamadan University of Medical Sciences, Hamadan, Iran

## Abstract

**Background:**

Gastrosphere, an enriched cellular population with stem-like properties believed to be responsible for an escape from immune-mediated destruction. Th17 and Treg cells play a major role in gastric cancer; however, their interaction with gastrospheres remained elusive.

**Method:**

Peripheral blood mononuclear cells were isolated from healthy donors and were cultured with conditioned media of MKN-45 (parental) cells as well as gastrospheres' conditioned media in the context of mixed lymphocyte reaction and in the presence of anti-CD3/CD28 beads. The proliferation was evaluated using CFSE staining; the percentages of CD4^+^CD25^+^FoxP3^+^ Treg and CD4^+^IL-17^+^ Th17 cells and IFN-*γ*+cells and the production of IL-17, TGF-*β*, and IL-10 were assessed by flow cytometry and ELISA, respectively. Finally, the cytotoxic potential of induced immune cells was measured by examining the secretion of lactate dehydrogenase from target cells.

**Results:**

The results revealed a decreased expansion of PBMCs postexposure to gastrospheres' conditioned medium which was concomitant with an increased percentage of Th17 and an enhanced Th17 to Treg ratio. The conditioned media of gastrospheres enhanced the secretion of IL-10 and IL-17 and decreased TGF-*β*. Interestingly, immune cells induced by gastrospheres showed significant cytotoxicity in terms of producing IFN-*γ* and death induction in target cells. All these changes were related to the upregulation of IL-6, IL-10, and IL-22 in gastrospheres compared to parental cells.

**Conclusion:**

Our study showed that the condition media of gastrospheres can potentially induce Th17 with increasing in their cytotoxic effect. Based on our knowledge, the present study is the first study that emphasizes the role of gastrospheres in the induction of antitumor Th17 cells. However, it should be confirmed with complementary studies *in vivo*.

## 1. Introduction

Gastric cancer stem cells (CSCs) are considered as the key player for the initiation and development of tumors which give rise to nontumorigenic and invasive tumor cells. They are responsible for metastasis and immune escape [1] and can be isolated and enriched by different mechanisms including stem cell surface markers, intracellular enzyme activity, the concentration of reactive oxygen species, the identification of side population cells, resistance to cytotoxic compounds or hypoxia, invasiveness/adhesion, immunoselection, and sphere formation in nonadherent conditions [2]. Gastrosphere is an *in vitro* tridimensional (3D) culture model of gastric CSCs with stemness properties [3].

In general, CSCs employ several mechanisms to evade the immune system such as impairment of antigen presentation to prevent cytotoxic T cell activation, downregulation of CD80 and upregulation of PDL-1 to induce T cell anergy, and induction of immunosuppressive M2 macrophages by the production of CSF, TGF-*β*, and MIC-1 in which in turn inhibits the induction of proinflammatory antitumor M1 macrophages. Furthermore, M2 macrophages impair the activation and proliferation of T cells through IL-10 and TGF-*β* secretion. They recruit and expand immune suppressive Treg cells to the tumor microenvironment [4].

In addition to Treg cells as a wholly immunosuppressive population, changes in Treg and Th17 paradigm have recently been taken into consideration in cancers [5, 6]. Recent studies suggest that both Th17 cells and FoxP3^+^ T cells are able to regulate antitumor responses negatively or positively depending on the microenvironment and type of cancer which have a remarkable effect on the number and function of these cells [7]. According to the previous data, the accumulation of Th17 and Treg cells in the gastric tumor microenvironment is associated with the clinical stage and leads to an imbalanced Th17/Treg in patients with advanced gastric cancer [8–10]. These studies demonstrated the distribution of Th17 cells in relation to Treg in peripheral blood, tumor-draining lymph nodes, and tumor tissues of patients with gastric cancer compared to healthy individuals [10, 11]. IL-6 and TGF-*β* induce Th17 differentiation either in normal condition [12, 13] or in gastric cancer that it leads to an imbalanced Th17/Treg.

Activation of gastric CSCs could be one of the candidates for the imbalanced Th17/Treg in advanced gastric cancer due to their secretions. More recently, it was shown that the presence of IL-17 in the tumor microenvironment of advanced gastric cancer is correlated with stemness upregulation [14] and transforms gastric CSCs into active ones [15]. This other point of view implies a reciprocal relationship between Th17 and gastric CSC activation. Due to the lack of enough data regarding the effect of gastric CSCs on the Th17/Treg paradigm, the present study was designed to further explore the relationship between CSCs and Th/Treg balance and their subsequences in tumor immunity in vitro. For this purpose, we investigated the frequency and the balance of Th17 and Treg cells in peripheral blood mononuclear cells postexposure to conditioned media derived from human gastric cancer cells and their enriched gastrospheres as a model for gastric CSCs.

## 2. Material and Methods

### 2.1. Parental Cell Culture and Sphere Formation

The human gastric cancer cell line (MKN-45) from a 62-year-old woman with poorly differentiated gastric adenocarcinoma (NCBI code: C615) was provided by the National Center for Genetic Resources of Iran. MKN-45 cells were cultured in Roswell Park Memorial Institute (RPMI) medium-1640 (Gibco, USA) supplemented with 10% fetal bovine serum (FBS, Gibco, USA), 100 U/ml penicillin, and streptomycin (Thermo Fisher, USA) as an adherent monolayer culture and were trypsinized to a single cell preparation.

In the following, sphere formation was performed to enrich cancer stem cells from parental cells [3, 16]. Briefly, sphere bodies were obtained by seeding MKN-45 cells at a density of 10^5^ cells/ml in serum-free RPMI supplemented with B27 2% (50X, Gibco, USA), 20 ng/ml of basic fibroblast growth factor (bFGF, Royan Biotech, Iran), and epidermal growth factor (EGF, Royan Biotech, Iran) in T-25 nonadhesive poly(2-hydroxyethyl methacrylate) (poly-HEMA, Sigma, USA) coated flasks. B27, bFGF, and EGF were refreshed every 48 hours. Sphere formation was examined using an inverted microscope at ×10 and ×20 magnifications. The gastrospheres were formed after 4-5 days and then were dissociated enzymatically with trypsin (Gibco, USA) into single cells and moved to other flasks to obtain secondary passage.

### 2.2. Conditioned Media Preparation

Appropriate density (about 70%) of monolayer cell culture and also the enriched gastric CSCs in passage two were dissociated in single cells and seeded in a concentration of 3 × 10^4^ cells/well in two separate poly-HEMA coated and uncoated 96-well plates for obtaining parental cells and gastric CSCs' conditioned media, respectively, in serum-free RPMI with a volume of 200 *μ*l at 37°C and 5% CO2. The conditioned media were collected and centrifuged at 400xg after 24 hours and were then filtered by 0.2 *μ*m filter to remove debris and transferred to 1.5 *μ*l microtubes for storage at -80°C. Parental cells and gastric CSCs were also collected in RNase-free tubes and stored at -80°C.

### 2.3. Peripheral Blood Mononuclear cell (PBMC) Preparation and General Expansion

PBMCs were isolated from two healthy volunteers using Hydroxyethyl Starch (HES 6%) (GRIFOLS, Spain) to remove red blood cells, followed by buffy coat isolation using Lymphodex (Inno-Train, Germany) density gradient centrifugation. Mixed lymphocyte reaction )MLR( was performed to obtain an appropriate ratio of PBMCs to secreting parental cells/gastric CSCs as follows. To inhibit the proliferation of the group that plays the role of a stimulator, a population of PBMCs was treated with mitomycin C at a final concentration of 10 *μ*g/ml within 60-90 minutes. Mitomycin C acts via the inhibition of DNA and RNA synthesis, rendering the lymphocytes unable to proliferate or activate. The PBMCs were washed three times with 5 ml complete medium to neutralize and remove mitomycin C traces and prepared as the stimulator. The responder and stimulator populations were cultured in the presence of parental cells and gastric CSCs' conditioned media at ratios 1 : 1, 1 : 5, and 1 : 10 with prepared parental and CSC conditioned media for 5 days. One, 5, and 10 correspond to PBMCs of the responder to the number of parental cells and CSCs whose conditioned media has been collected. A group with a fresh medium was considered as the control group.

To stimulate T cell expansion through the first and second signal activation, PBMCs isolated from healthy individuals and were stimulated by anti-CD3/CD28 microbeads (Dynabeads Human T-Activator, Gibco, USA) based on the company's instructions in the presence of conditioned media of parental cells and gastric CSCs in a ratio of 1 : 5 for 5 days. A group with a fresh medium was also considered as a control group. IL-2 (Royan Biotech, Iran) was used for T cell proliferation in 30 IU/ml concentration.

To evaluate the PBMCs' proliferation in the study groups, responder populations were prestained with CFSE (carboxyfluorescein succinimidyl ester, Invitrogen, USA) in each group, according to the company's instructions, and analyzed using a flow cytometer (BD FACSAria, USA) at the first and last days of culture.

### 2.4. Immunophenotyping

PBMCs were resuspended at a density of 1 − 1.5 × 10^5^/100 *μ*l in phosphate-buffered saline (PBS) and were incubated with surface antigen markers APC-labeled anti-CD4 (BD, USA) and FITC-labeled anti-CD25 (AbD Serotec, UK) for 30 minutes at 4°C in the dark. To fix the surface markers and increase the permeability of the membrane, Cytofix/Cytoperm™ solution (BD, USA) was applied as per manufacturer's instruction and placed at 4°C. After 20 minutes, PBMCs were washed twice using a Perm/Wash™ (1X) (BD, USA) solution. Cells in each group were incubated with the intracellular antibodies PE-labeled anti-FoxP3 (Biolegend, USA) and PE-labeled anti-IL-17 (Invitrogen, USA) in the dark for 60-45 minutes and then washed increase using Perm/Wash™ (1X) to analyze by flow cytometer.

### 2.5. Enzyme-Linked Immunosorbent Assay

To measure the concentrations of IL-17a, IL-10, and TGF-*β* in PBMC's supernatant, an enzyme-linked immunosorbent assay (ELISA) was performed using commercially available kits for cytokine detection (R&D systems for human IL-17a, IL-10, and TGF-*β*, USA). The preparation of all reagents, the working standards, and protocol were followed according to the manufacturer's instructions. The absorbance was read using an ELISA reader (BIO-RAD, UK) at 450 nm and 570 nm dual filters. The detection ranges for IL-17a and TGF-*β* were 31.2-2,000 pg/ml, and for IL-10 were 7.8-500 pg/ml. All the samples were thawed only once.

### 2.6. LDH Release Assay

MKN-45 cells were plated at a density of 3 × 10^4^ cells per well in 96-well culture plates and incubated for 24 h as target cells. PBMCs postculture of parental cells and gastrospheres' conditioned media were collected and then added to each well in the ratio of 1 : 3 (3 corresponds to PBMCs to target MKN-45 cells) and incubated at 37°C for a further 72 h. One group consisted of MKN-45 cells in the absence of PBMCs, and one group consisted of MKN-45 cells containing 20% Triton were considered as negative and positive control, respectively. A colorimetric assay was applied according to the LDH assay kit (Pars azmoon, Iran), and then, the content of LDH released from the cells to the culture medium was calculated according to the recipe kit.

### 2.7. RNA Extraction and qRT-PCR

Parental MKN-45 cells and their derived gastrosphere in passage two were collected and stored at -80°C until RNA extraction. Total RNA was isolated using Trizol reagent (Qiagen, USA). The quality of RNA samples was assessed by agarose gel electrophoresis and a spectrophotometer (Biowave ІІ, UK). A total of 2 *μ*g of RNA was reverse transcribed with a cDNA synthesis kit (Takara) according to the manufacturer's instructions. Transcript levels were determined using the SYBR Green master mix (Takara, Japan). Primer sequences for quantitative real-time polymerase chain reaction (qRT–PCR) are listed in Table 1. Expression of genes involved in the differentiation of Th17 and Treg was normalized to the *GAPDH* housekeeping gene. Relative quantification of gene expression was calculated using the *ΔΔ*Ct method.

### 2.8. Statistical Analysis

Statistical analyses were carried out using the GraphPad Prism 6.01 statistical software. Data were presented as mean ± SD for 3 replicates. Significant differences among mean values were evaluated by two-way ANOVA for T cells' colony size, proliferation index, and qRT-PCR, one-way ANOVA for phenotyping and LDH assays, and the Student *t*-test for comparison of Th17 to Treg ratio between groups. *P* < 0.05 was considered as statistically significant.

## 3. Results

### 3.1. Gastrospheres Inhibit PBMC Expansion

**Figure 1:**
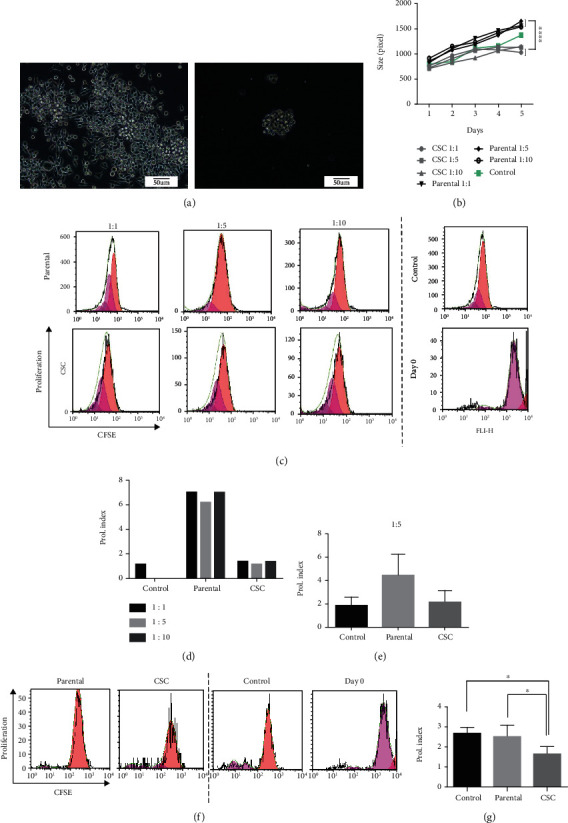
Gastrospheres' conditioned medium limits PBMC proliferation. (a) Monolayer culture of MKN-45 gastric cancer cell line and its derived-loose grape-like shape gastrospheres. (b) Colony size of PBMCs in exposure to MKN-45 parental cells or gastrosphere-derived conditioned media in MLR. (c, d) PBMCs' proliferation in exposure to parental or gastrospheres' conditioned media in MLR (*n* = 1). (e) PBMCs' proliferation in exposure to parental or gastrospheres' conditioned media in MLR at ratio 1 : 5. (f, g) PBMCs' proliferation in exposure to parental or gastrospheres' conditioned media in the presence of anti-CD3/CD28 microbeads at ratio 1 5. CSC: gastrospheres. Data are mean ± SD of three independent experiments. ^∗^*P* < 0.05.

Our previous studies determined the stem-like properties of gastrospheres [3]. Therefore, in the present study, we used gastrospheres (Figure 1(a)) as a model of gastric CSCs in comparison to their parental cells. To evaluate the effect of gastric-CSCs on the immune cells on peripheral blood, the conditioned media of gastrospheres and parental cells were used. Our results depicted that the secretion of gastrospheres caused a reduction in colony size (*P* = 0.0001, Supplementary Fig 1, Figures 1(a) and 1(b)) and cell proliferation index (*P* = 0.7, Figures 1(c)–1(e)) of PBMSCs at all different ratios. Moreover, CFSE proliferation assay revealed a reduction in stimulated PBMCs with anti-CD3/CD28 microbeads posttreatment of gastrospheres' conditioned media at the ratio of 1 : 5 (*P* = 0.04, Figures 1(f) and 1(g)). All these changes may be related to the promotion of cell differentiation in PBMSCs posttreatment with gastric CSCs secretome.

### 3.2. Gastrospheres Have a Distinct Effect on Th17 and Treg Differentiation

**Figure 2:**
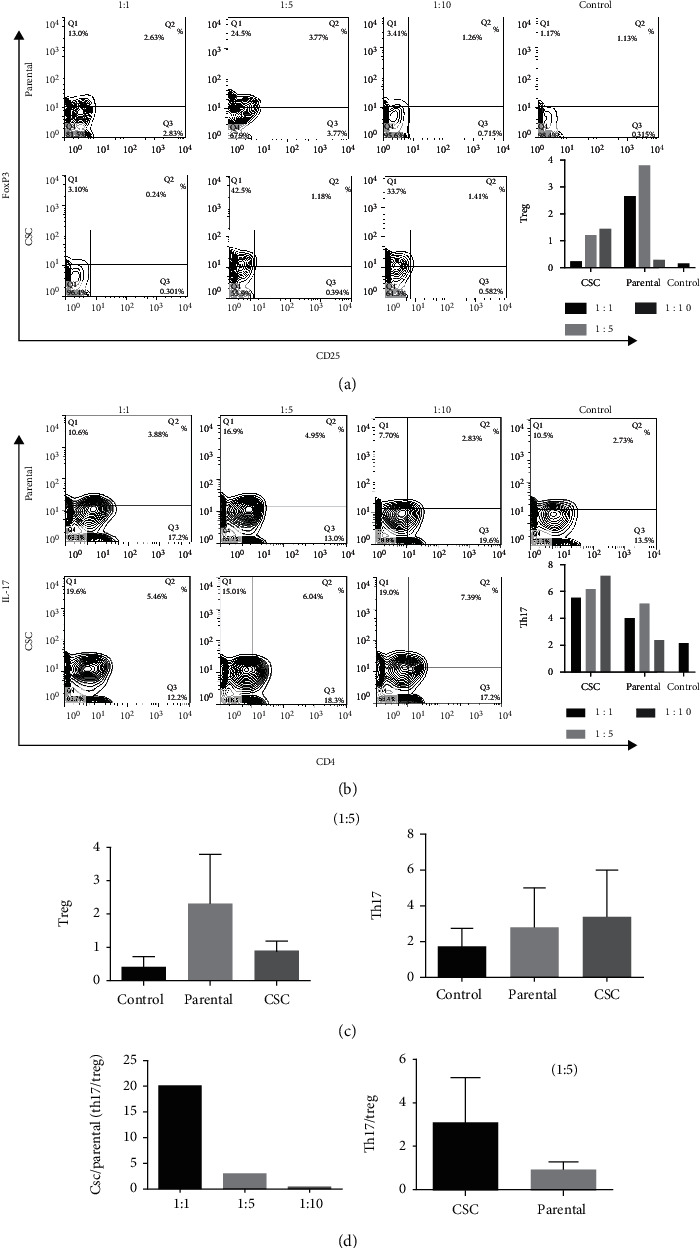
Differentiation of Th17 and Treg in exposure to parental cells and gastrosphere-derived condition media in MLR. (a, c) CD4^+^CD25^+^FoxP3^+^ Treg cell differentiation after exposure to gastrospheres or parental-derived condition media. (b, c) CD4^+^IL-17^+^ Th17 cell differentiation after exposure to gastrospheres or parental-derived condition media. (d) The ratio of Th17 to Treg after exposure to gastrospheres or parental-derived condition media. CSC: gastrospheres. Data are mean ± SD of three independent experiments.

**Figure 3:**
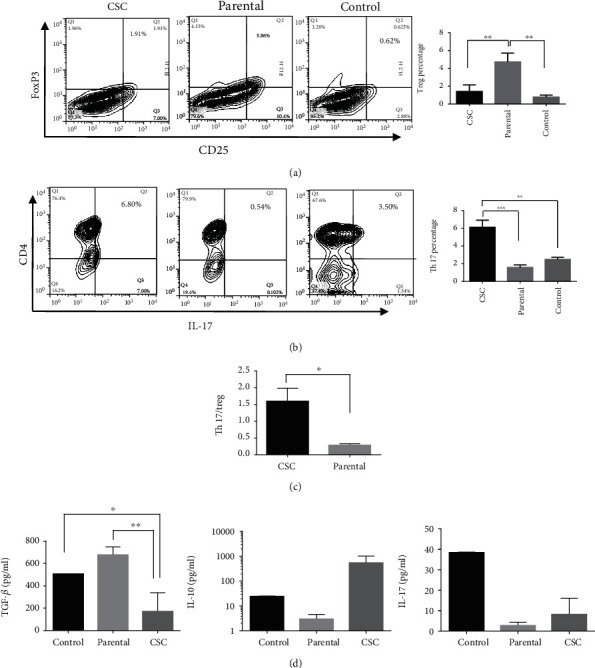
Differentiation of Th17 and Treg after exposure to gastrospheres and parental-derived condition media in the presence of anti-CD3/CD28 microbeads. (a) CD4^+^CD25^+^FoxP3^+^ Treg cell differentiation after exposure to gastrospheres or parental cell-derived condition media. (b) CD4^+^IL-17^+^ Th17 cell differentiation after exposure to gastrospheres or parental cell-derived conditioned media. (c) Th17 to Treg ratio after exposure to gastrospheres or parental cell-derived conditioned media. (d) Concentrations of IL-17, TGF-*β*, and IL-10 cytokines measured by ELISA in supernatants of PBMCs after being exposed to gastrospheres or parental cells' conditioned media. CSC: gastrospheres. Data are mean ± SD of three independent experiments. ^∗^*P* < 0.05, ^∗∗^*P* < 0.01, and ^∗∗∗^*P* < 0.001.

To understand how gastric CSCs affect Th17 and Treg cell differentiation, the conditioned media of gastrospheres and parental cells were added to the culture media of MLR or in the setting of T cell proliferation with anti-CD3/CD28 microbeads. Our data revealed that gastrospheres' secretions not only increased the levels of Th17 (*P* = 0.9) but also enhanced the ratio of Th17 to Treg cells in the MLR (Figures 2(a)–2(d)) and even when T cells cultivated in the presence of anti-CD3 and CD28 microbeads (*P* = 0.0008, Figure 3(b)). However, the parental cell secretions just promoted the level of Treg cells (*P* = 0.5) in the MLR setting (Figure 2(a)) and even in PBMCs which were stimulated by anti-CD3 and CD28 microbeads (Figure 3(a)). Importantly, the ratio of Th17 to Treg and the concentrations of IL-17 and IL-10 in the presence of gastrospheres' conditioned medium were higher than of the parental cells' conditioned medium (Figures 3(c) and 3(d)). In contrast, TGF-*β* level which is the most important cytokine produced by Treg cells was notably higher in the supernatant of PBMCs treated with parental cells' conditioned medium (*P* = 0.0024, Figure 3(d)).

### 3.3. Mediators Expressed by Gastrospheres Are Involved in Th17/Treg Balance

**Figure 4:**
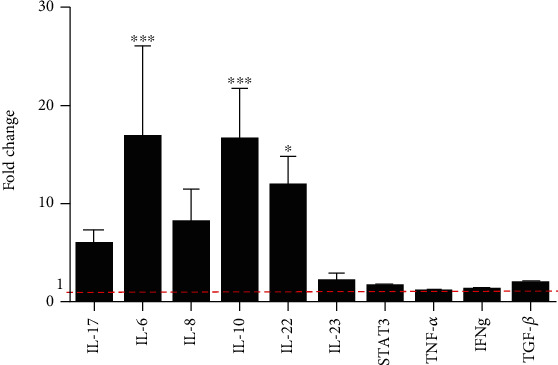
Gene expression of IL-17, IL-6, IL-8, IL-10, IL-22, IL-23, STAT3, TNF-*α*, and TGF-*β* in gastrospheres compare to parental cells. Data are mean ± SD of four independent experiments. ^∗^*P* < 0.05, ^∗∗^*P* < 0.01, and ^∗∗∗^*P* < 0.001.

To further understand that how gastric CSCs could affect Th17/Treg balance, we evaluated the expression of IL-17, IL-6, IL-8, IL-10, IL-22, IL-23, TNF-*α*, TGF-*β*, IFN-*γ*, and STAT3 that believed to be important in changing the Th17/Treg balance using qRT-PCR in parental and gastrospheres. Our results revealed a significant increase in the expression of IL-6 (*P* = 0.0007) which is crucial in changing the balance towards Th17, as well as IL-10 (*P* = 0.0009) and IL-22 (*P* = 0.0478) in gastrospheres compared to parental cells (Figure 4).

### 3.4. Gastrospheres Induced Cytotoxic Immune Populations

**Figure 5:**
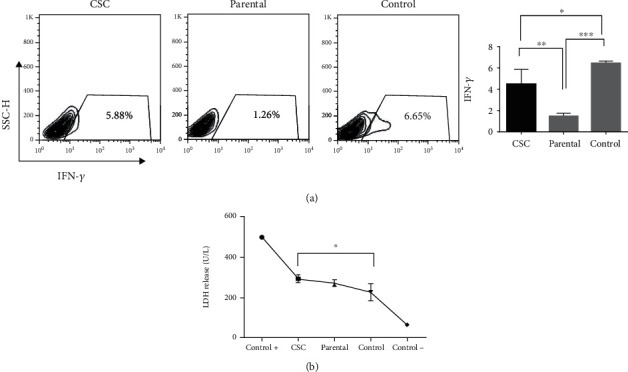
Cytotoxicity of T cells after exposure to gastrospheres and parental cell-derived conditioned media. (a) IFN-*γ* levels in PBMCs after exposure to gastrospheres or parental cell-derived conditioned media. (b) LDH release of MKN-45 cells (target) in culture with gastrospheres or parental conditioned media induced lymphocytes (effector). CSC: gastrospheres. Data are mean ± SD of three independent experiments. ^∗^*P* < 0.05, ^∗∗^*P* < 0.01, and ^∗∗∗^*P* < 0.001.

To assess the function of gastric CSC-induced immune cells, we investigated the production of IFN-*γ* in PBMCs posttreatment with conditioned media of gastrospheres and parental cells. Interestingly, we observed an increase in the level of IFN-*γ* following treatment with gastrospheres' secretion (*P* = 0.009, Figure 5(a)). To further understand the function of induced immune cells by the conditioned media of gastrospheres and parental cells, we also examined the cytotoxicity of induced immune cells as effector cells in direct culture with MKN-45 gastric cancer cell line as target cells. The toxicity was assessed by the target cell release rate of LDH. LDH is a cytoplasmic enzyme retained by viable cells with intact plasma membranes but released from necrotic cells with damaged membranes. Our results showed a high concentration of LDH in the supernatant derived from the interaction of gastrosphere-induced immune cells and target cells (*P* = 0.02, Figure 5(b)). These data suggested that gastrosphere-induced immune cells had an antitumor effect by killing MKN-45 cells. We further examined the possible existence of mature dendritic cells in experimental groups. Our results showed that gastrosphere-derived conditioned medium induced mature dendritic cells expressing CD11c and CD80 markers among PBMCs, while similar results were not observed in PBMCs treated with parental cells' conditioned medium (data not shown).

## 4. Discussion

CSCs are responsible for the tumor genesis, metastasis, and recurrence of cancers. Identifying these cells in different cancers and introducing their distinctive features in comparison with other tumor cells, especially their effect on the immune system, can be promising targets in therapies. The tumor microenvironment can invoke and induce Th17 and Treg cells and change the Th17/Treg balance in advanced and metastatic gastric cancer [10]. Nevertheless, how the cells among the tumor mass are responsible for the induction and disturbing the balance between Th17 and Treg yet to be determined. In this study, we intended to answer the question that whether there is a difference in inducing a change in the balance of Th17/Treg due to the factors secreted by gastric parental cells and gastric CSCs. For this purpose, gastrospheres were used as a model due to stemness properties, which we proved in our previous study [3].

Our results showed that the conditioned medium of gastrospheres decreased the proliferation of PBMCs, even if the PBMCS stimulated with anti-CD3/CD28 microbeads as a strong stimulator of T cell expansion. This suggested that the secretome of gastrospheres can inhibit immune cell expansion. Although there is no evidence on decreasing T cell expansion by gastric CSCs, it has been reported that tumor cells, directly and/or indirectly, limit CD4^+^ and CD8^+^ T cell expansion, function, and memory formation in many cancers including gastric cancer [17, 18]. Conversely, the conditioned medium of parental cells increased the proliferation of PBMCs. Accumulating data have shown an increased frequency of T cells peripherally and locally by different mechanisms in several cancers [19–21]. A possible mechanism for the increase of lymphocyte proliferation index could be the recognition of antigens by lymphocytes especially by T cells or the presence of soluble factors that induce an increase in lymphocyte proliferation in parental cell secretome.

Currently, Treg cells are known to be the main subset of immune cells that increased during cancer progression. Several types of tumor cells can also recruit Treg cells into the tumor site by their secretions [22]. Our results indicated that the conditioned medium of gastrospheres derived from gastric cancer cells can increase Th17 frequency. We also observed that gastrospheres skewed Th17/Treg balance toward Th17 differentiation through their secretion. Despite insufficient data on disturbance of Th17/Treg balance in cancers, previous reports determined the induction of Th17 producing IL-10 cells in vivo, in vitro, and in gastric cancer patients [10, 23]. Moreover, many studies also have reported the increase of Treg cells within the tumor microenvironment of gastric cancer patients [10, 24]. It seems that the accumulation of Th17 and Treg cells in the tumor microenvironment following disease progression leads to an imbalance in Th17/Treg cells in cancers including advanced gastric cancer [10, 25, 26]. Previous studies have reported a concomitant increase in Th17 cells and the metastasis of gastric cancer [25, 27]. Another recent study showed a decreased number of Th17 cells despite an increase in the number of Treg cells accompanied by an increased expression of the immunosuppressive axis of PD-1/PD-L1 in patients who underwent gastric cancer resection. In this study, silencing PD-1 has been shown to alter the Th17/Treg toward Th17 cells [28].

Soluble mediators and cell to cell communications are two main factors enabling CSCs to induce th17 differentiation [29]. Moreover, CSCs seem to have an important role in changing the balance of Th17 and Treg cells in advanced gastric cancer. Our findings suggest that the expression of IL-6 by gastrospheres as a game-changer in the expression of FoxP3 or ROR*γ* contributes to the differentiation of Th17 and changing the balance of Th17/Treg. IL-6 is an important mediator which secretes from cancer cells in high concentrations and highly promotes tumorigenesis and protects the cancer cells from therapy-induced DNA damage, oxidative stress, and apoptosis by inducing several pathways [30]. Besides, it prevents apoptosis and skews naïve CD4^+^ T cells towards proinflammatory Th17 by inhibiting TGF-*β*-driven expression of Foxp3 [12, 31, 32]. Similar to our results, higher production of IL-6 has been reported in CSCs of head and neck squamous cell carcinoma [33]. Moreover, there is evidence that secretion of IL-10 from cancer stem cells reduces the proliferation of T cells and promotes tumor progression [34, 35]. Furthermore, increased level of intratumoral and circulating IL-22 have been found in gastric cancer patients and are associated with cell survival, migration, proliferation, and angiogenesis [36]. Accordingly, higher expression of IL-6, IL-10, and IL-22 by gastric CSCs, along with the immunoregulatory effects, be primarily is in favor of gastric CSCs themselves and may contribute to maintaining stemness and self-renewal of CSC cells by an autocrine effect [37].

Interestingly, in the present study, we presented data that gastric CSCs' conditioned medium can induce cytotoxicity in PBMCs through increasing of IFN-*γ* producing cells and induction of necrosis in cancer cells. It seems that IL-17^+^/IFN-*γ*^+^ cells exert a strong antitumor effect *in vitro* and *in vivo* [38]. Antitumor activity of a new population of IL-17^+^/IFN-*γ*^+^ CD8 T cells (known as Tc17 cells) has also been reported in some cancers, and interestingly, noncytotoxic Tc17 cells can become cytotoxic in the presence of IL-12. These studies show that the polarity and functions of the IL-17^+^ subset depend on the cytokine profile in the tumor microenvironment [39].

In conclusion, our findings suggest that gastrospheres as a model of gastric CSCs affect immune cells differently than the cancer cells. They secret different factors that (1) potentially affect the plasticity and the balance between Th17 and Treg cells, (2) possibly induce IFN-*γ*-producing T cells with antitumor properties, and (3) help to maintain self-renewal properties in gastrospheres. However, this data should be confirmed by other experiments.

## Figures and Tables

**Table 1:** Primer sequences used for quantitative real-time polymerase chain reaction.

Primer name	Primer sequence
IL-17	F: 5′ AACCGATCCACCTCACCTTG 3′
	R: 5′ CCCACGGACACCAGTATCTT 3′
IL-6	F: 5′ A G G A G A C T T G C C T G G T G A AA 3′
	R: 5′ C A G GGG T G G T T A T T G C A T C T 3′
IL-8	F: 5′ T A G C A AAA T T G A G G C C A A G G 3′
	R: 5′ A G C A G A C T A G GG T T G C C A G A 3′
IL-10	F: AAG CTG AGA ACC AAG ACC CA
	R: AAG GCA TTC TTC ACC TGC TC
IL-22	F: TGTGAGCTCTTTCCTTATGG
	R: TGCGGTTGGTGATATAGGGC
IL-23	F: GCAGATTCCAAGCCTCAGTC
	R: CCTTGAGCTGCTGCCTTTAG
STAT3	F: 5′ GAAGAATCCAACAACGGCAG 3′
	R: 5′ TCACAATCAGGGAAGCATCAC 3′
TNF-*α*	F: 5′ CCTCTCTCTAATCAGCCCTCTG 3′
	R: 5′ GAGGACCTGGGAGTAGATGAG 3′
IFN-*γ*	F: 5′ GGTTCTCTTGGCTGTTACTG 3′
	R: 5′ TCTTTTGGATGCTCTGGTCA 3′
TGF-*β*	F: 5′ AACCCACAACGAAATCTATGAC 3′
	R: 5′ TAACTTGAGCCTCAGCAGAC 3′

IL-17: interleukin 17; IL-8: interleukin 8; IL-6: interleukin 6; IL-10: interleukin 10; IL-22: interleukin 22; IL-23: interleukin 23; STAT3: signal transducer and activator of transcription 3; TNF-*α*: tumor necrosis factor alpha; IFN-*γ*: interferon gamma; TGF-*β*: transforming growth factor beta.

## Data Availability

(1) The data (original) used to support the findings of this study are included within the article. (2) The data (original) used to support the findings of this study are included within the supplementary file.

## Supplementary Materials

Supplementary Materials Supplementary Figure 1: T cell colonies under treatment of parental and gastrospheres' conditioned media in ratios of 1 : 1, 1 : 5, and 1 : 10 in MLR. CSC: gastrospheres; CM: conditioned medium.
