# Supplementary figures and images for: Gastrospheres as a Model of Gastric Cancer Stem Cells Skew Th17/Treg Balance toward Antitumor Th17 Cells
Source: J Immunol Res. 2020 Dec 23;2020:6261814. doi: 10.1155/2020/6261814 (PMC7775146; doi:10.1155/2020/6261814)

a

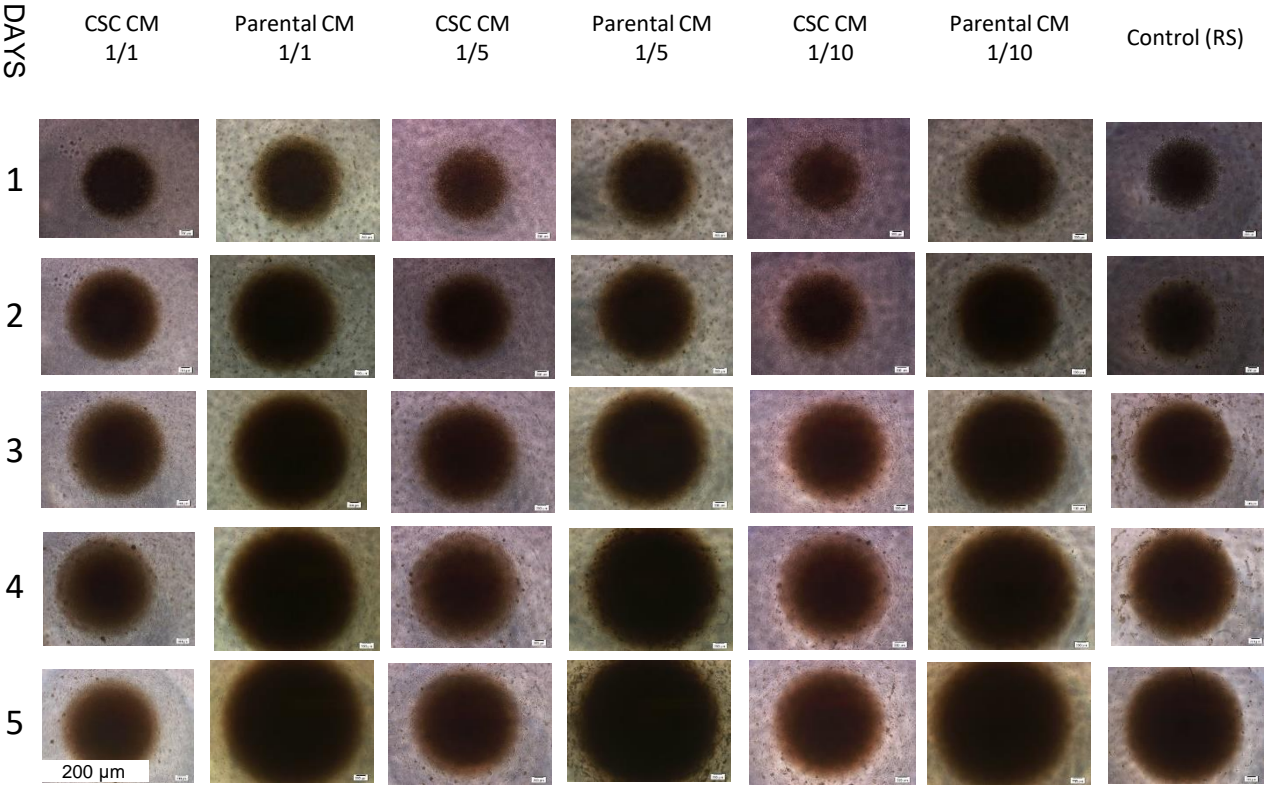

Supplement: Supplementary Materials — Supplementary Figure 1: T cell colonies under treatment of parental and gastrospheres' conditioned media in ratios of 1 : 1, 1 : 5, and 1 : 10 in MLR. CSC: gastrospheres; CM: conditioned medium. [file 6261814.f1.pdf]
